# The Perspectives of Plant Natural Products for Mitigation of Obesity

**DOI:** 10.3390/nu15051150

**Published:** 2023-02-24

**Authors:** Daotong Li, Fang Chen

**Affiliations:** National Engineering Research Center for Fruit and Vegetable Processing, Key Laboratory of Fruits and Vegetables Processing, College of Food Science and Nutritional Engineering, Ministry of Agriculture, Engineering Research Centre for Fruits and Vegetables Processing, Ministry of Education, China Agricultural University, Beijing 100083, China

Obesity is a metabolic disease caused by an imbalance between energy intake and consumption, which leads to excessive fat accumulation in adipose tissues. The prevalence of obesity is increasing worldwide and its associated metabolic disorders have alarmingly become a global public health issue that severely affects the health and quality of life of people [1]. It is estimated that two billion people (nearly one-third of the world’s population) have been classified as obese [2]. The obesity-associated hypertension, hyperglycemia and dyslipidemia are also risk factors that are closely linked to multiple diseases such as diabetes mellitus, cardiovascular disease, and cancer, which are major causes of death worldwide [3]. The rapid increase in the prevalence of obesity and the associated metabolic dysfunction highlights the urgent need for managing the detrimental health effects. Weight loss by surgical treatment is effective in reducing the risk of morbidity and mortality, while it may cause negative emotions in people. Some anti-obesity drugs have been available and approved for the treatment of obesity, whereas their use may trigger certain side effects [4]. Exploring and developing risk-free and efficient weight management treatments to prevent and treat obesity is still an unmet clinical need.

Recently, plant-derived natural compounds have attracted much attention due to their relatively high safety profile with fewer side effects. In our Special Issue entitled “The Perspectives of Plant Natural Products for Mitigation of Obesity”, several articles have been published that focus on the crucial role of plant natural products in mitigating obesity. Flavonols are a group of flavonoids that include quercetin, kaempferol, myricetin, and isorhamnetin. Onions, tea, apples, kale, lettuce, tomatoes, broccoli, and grape skins are important foods of dietary flavonols. By investigating the relationship between dietary flavonols intake and obesity parameters (fat mass, waist circumference, and body mass index) in 40 obese and 40 healthy volunteers, the study by Joanna Popiolek-Kalisz [5] provided evidence that consumption of flavonol-enriched foods may play a protective role in obesity development. Kochujang is a Korean fermented, soybean-based red pepper paste that has been reported to have obesity-reducing effects in rats. In a randomized, double-blind clinical trial, the study of Han et al. [6] compared the anti-obesity effects of traditional Kochujang and commercially available Kochujang in overweight and obese patients. The results showed that the traditional Kochujang is superior to the commercial Kochujang in reducing body fat content and improving blood lipid profiles. These two human studies suggest that long-term consumption of functional foods enriched in natural dietary phytochemicals is effective to reduce the risk of obesity and metabolic syndrome.

The development of obesity is closely associated with adipogenesis, which is characterized by an increased number and size of adipocytes in adipose tissues. The differentiation of preadipocytes into mature adipocytes is a key step for cellular lipid metabolism and accumulation [7]. Inhibition of adipogenesis and lipid accumulation in adipocytes may be an effective strategy for regulating obesity. Warinhomhoun et al. [8] isolated the natural metabolites from an orchid species in Thailand and evaluated their biological impact on the growth and differentiation of mouse embryonic 3T3-L1 pre-adipocytes. They found that a bibenzyl compound could suppress adipogenesis by inhibiting adipocyte differentiation and lipid accumulation. The underlying molecular mechanisms were linked to the downregulation of adipogenic regulators such as peroxisome proliferators activated receptor γ (PPARγ) and CCAAT/enhancer-binding protein α (C/EBPα) and the suppression of mitogen-activated protein kinase (MAPK) pathway. Of note, the plant natural compounds can be used as a mixture to exert synergistic effects for the prevention and treatment of obesity. The study by Lee et al. [9] reported that a combination of *p*-synephrine, *p*-octopamine HCl, and hispidulin effectively reduced the expression of adipogenic marker proteins (PPARγ, C/EBPα, and C/EBPβ) in 3T3-L1 adipocytes and mitigated the body weight gain in high-fat diet (HFD)-fed mice. Xu et al. [10] explored the anti-obesity effect and mechanism of nuciferine, a main aporphine alkaloid component in lotus leaf, in HFD-fed mice. They found that treatment of nuciferine prevented body weight gain, improved glycolipid metabolism, and promoted energy expenditure in obese mice. Nuciferine suppressed lipogenesis and promoted fatty acid oxidation by activating the AMP-activated protein kinase (AMPK) pathway in hepatocytes and adipocytes. Liquiritigenin is a natural flavonoid isolated from the herb Glycyrrhiza uralensis Fisch. Qin et al. [11] studied the effects of liquiritigenin on lipogenesis in adipocytes and the results showed that liquiritigenin was able to inhibit lipid accumulation in 3T3-L1 cells via a mammalian target of rapamycin (mTOR)-mediated autophagy mechanism. Polymethoxyflavones are flavonoids found in citrus fruits with bioactive properties. Jin et al. [12] reported that the polymethoxyflavones isolated from citrus leaves effectively improved HFD-induced insulin resistance and dyslipidemia in obese mice. The polymethoxyflavones exert the anti-obesity effect via the modulation of fatty-acid oxidation and lipolysis. Fennel is a herbaceous and perennial plant cultivated in China and fennel essential oil contains volatile natural compounds including *p*-anisaldehyde, limonene, estragole, anethole, and trans-anethole. The study by Hong et al. [13] reported that inhalation of fennel essential oil exerted a protective effect against lipid and metabolic dysfunction in HFD-induced obese rats.

Overall, this Special Issue provides compelling evidence of the role of plant-derived natural compounds in the treatment and prevention of obesity. Studies are required to further explore the potential pharmacological and physiological activities of bioactive phytochemicals and understand their underlying mechanisms of action.
